# ZnO-porous silicon nanocomposite for possible memristive device fabrication

**DOI:** 10.1186/1556-276X-9-437

**Published:** 2014-08-27

**Authors:** Lizeth Martínez, Oscar Ocampo, Yogesh Kumar, Vivechana Agarwal

**Affiliations:** 1 Center for Engineering and Applied Sciences (CIICAp-UAEM), Av. Universidad 1001. Col. Chamilpa, Cuernavaca, Morelos 62209, Mexico

**Keywords:** Memristor, Zinc oxide, Porous silicon, Composite, Nanocrystallites

## Abstract

Preliminary results on the fabrication of a memristive device made of zinc oxide (ZnO) over a mesoporous silicon substrate have been reported. Porous silicon (PS) substrate is employed as a template to increase the formation of oxygen vacancies in the ZnO layer and promote suitable grain size conditions for memristance. Morphological and optical properties are investigated using scanning electron microscopy (SEM) and photoluminescence (PL) spectroscopy. The proposed device exhibits a zero-crossing pinched hysteresis current-voltage (*I*-*V*) curve characteristic of memristive systems.

## Background

The memristor, known as the fourth fundamental circuit element, is a device whose main characteristic is the dependance of resistance according to the flux of charge passing through it and has the ability to remember its last resistance state. It was hypothesized by Chua [1] in 1971, but it was not until 2008 that it was first fabricated at HP Labs [2]. Since then, the fabrication and study of memristive devices have become very popular due to their applications in information storage, non-volatile memories, neural networks, etc. [3-5] Memristive switching behavior has been observed in many metal oxides [6,7] and attributed to the migration of oxygen vacancies within the oxide layers and grain boundaries [8,9], but still, transport mechanisms are being studied and different models have been suggested [7-9]. Zinc oxide (ZnO) possesses several interesting properties and has been extensively studied for its technological applications, specifically in electronic and optoelectronic devices such as photodetectors [10,11], light-emitting diodes [12], solar cells [13,14], and gas sensing [15]. On the other hand, porous silicon (PS)-ZnO composites have been used for white light emission [16] and to tune ZnO grain size for possible sensing applications [17]. This leads to the possibility to fabricate a tunable memristive device made of ZnO deposited on a PS template for optimizing the conditions of grain size, oxygen vacancies, defects, etc. to achieve tunable response from the device. The memristive behavior is demonstrated and explained through scanning electron microscopy (SEM) and photoluminescence (PL) characterization. The effect of annealing on morphology and photoluminescence response is also studied.

## Methods

PS samples were obtained by wet electrochemical etching using p^++^-type (100) Si wafers with a resistivity of 0.002 to 0.005 Ω cm. The anodization process was carried out using an electrolyte solution composed of hydrofluoric acid (48 wt% HF) and ethanol (99.9 %) in a volumetric ratio of 1:1. The bilayer porous structure was fabricated with a current density of *J*_1_ = 31.64 mA/cm^2^ (refractive index, *n*_1_ = 1.5) and *J*_2_ = 13.3 mA/cm^2^ (refractive index, *n*_2_ = 1.8). ZnO thin films were deposited on PS using sol-gel spin coating. In this process, zinc acetate dehydrate [Zn(CH_3_COO)_2_ · H_2_O] was first dissolved into the ethanol solution along with monoethanolamine (MEA). A homogeneous transparent solution with a concentration of 0.2 M zinc acetate and a 1:1 molar ratio of MEA/zinc acetate dehydrate was prepared. This solution was kept for hydrolysis for 48 h and spin coated onto the PS substrate seven times to get the desired film thickness. In order to study the stability and the good quality of ZnO, thin films were deposited on a Corning glass substrate (Corning Inc., Corning, NY, USA) and the transmittance measurements were taken with a PerkinElmer UV-Vis-NIR (Lambda 950) spectrophotometer (PerkinElmer, Waltham, MA, USA). To study the effect of annealing on the morphology of the ZnO film, samples were annealed in air atmosphere at 700°C for 30 min inside a tubular furnace. The orientation and crystallinity of the ZnO crystallites were measured by an X-ray diffraction (XRD) spectrometer (X'Pert PRO, PANalytical B.V., Almelo, The Netherlands) using CuKα radiation having a wavelength of 1.54 Å. The morphological effect of ZnO thin films with annealing was analyzed with a scanning electron microscope. The PL studies were carried out using a Varian fluorescence spectrometer (Cary Eclipse, Varian Inc., Palo Alto, CA, USA) under 3.8-eV excitation of a xenon lamp. The effect of the PS substrate on the electrical properties of the device (ZnO-PS) was studied by the acquisition of current-voltage curves applying DC voltage in a cyclic scan (from −10 to 10 V) at room temperature. Contacts were made of conductive carbon in two different configurations: lateral and transversal. A reference sample was fabricated and characterized by depositing ZnO on crystalline silicon.

## Results and discussion

**Figure 1:**
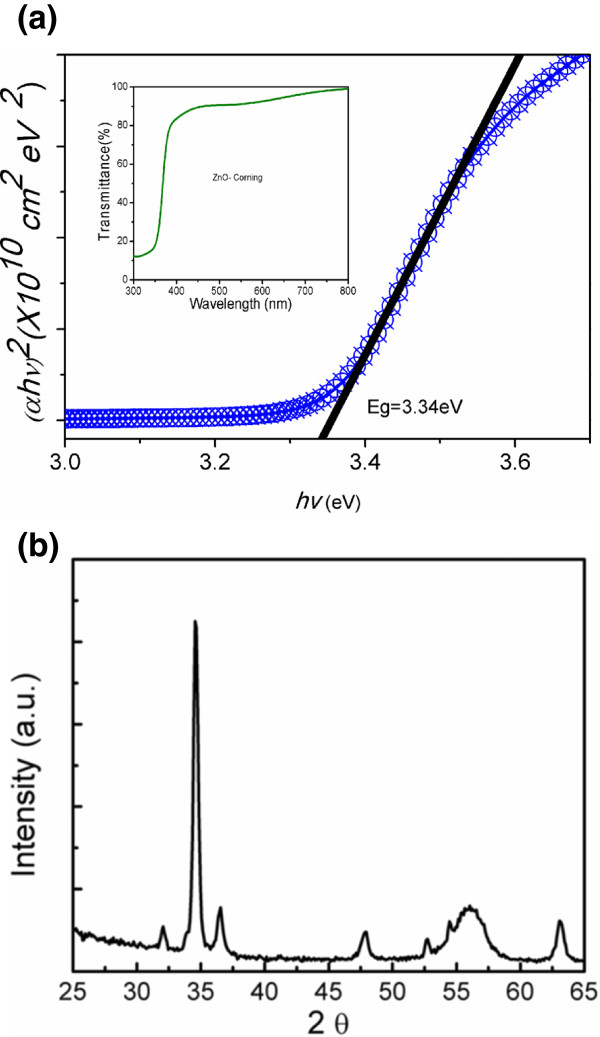
**Tauc plot and X-ray diffraction pattern. (a)** Tauc plot: optical absorption coefficient (*αhv*)^2^ vs. phonon energy (*hv*) of the ZnO thin film deposited on the Corning glass substrate. The inset shows the optical transmittance of the ZnO thin film on the Corning substrate. **(b)** X-ray diffraction pattern of the ZnO film after annealing at 700°C.

To check the quality of the ZnO film, its transmittance properties were analyzed as shown in (Figure 1) [18]. The absorption coefficient (*α*) is obtained using the following equation:

$$\alpha =-\frac{1}{d}\ln T$$

where *T* is the optical transmittance and *d* is the thickness of the ZnO thin film. For direct bandgap semiconductors, *E*_g_ can be estimated using the equation (*αhv*)^2^ = *A*(*hv* − *E*_g_), where *h* is the Planck constant, *v* is the frequency of incident photon, *A* is a constant, and *E*_g_ is the optical gap. Figure 1 shows the Tauc plot: (*αhv*)^2^ vs. phonon energy (*hv*) for measuring the direct bandgap of ZnO (3.34 eV) [19].

Figure 1b shows a typical XRD pattern (corresponding to the ZnO-PS structure annealed at 700°C). The graph exhibits the prominent peaks at 2*θ* = 32.0°, 34.61°, and 36.58° corresponding to the (100), (002), and (101) planes of ZnO, respectively. The XRD pattern of ZnO shows a hexagonal wurtzite structure and polycrystalline nature (JCDPS card number: 36-1451). The films are oriented perpendicular to the substrate surface in the *c*-axis. The *c*-axis orientation can be understood due to the fact that the *c*-plane of zinc oxide crystallites corresponds to the densest packed plane.

**Figure 2:**
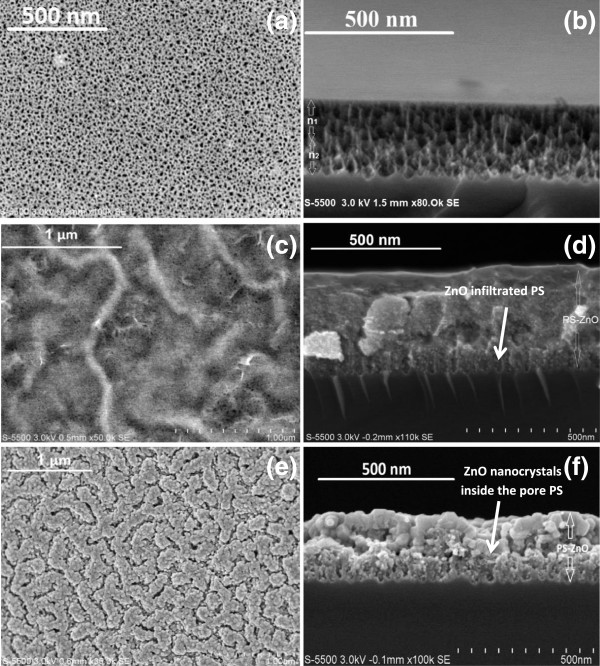
**SEM micrographs.** SEM micrographs show the top view of **(a)** PS substrate S1, **(c)** ZnO/PS composites ZS1, and **(e)** ZnO/PS composites after annealing at 700°C. **(b, d, f)**: Respective cross-sectional view of each sample.

Figure 2a shows the SEM image of the surface of the PS nanostructure (S1) with irregular distribution of pores. The average pore size is 20 nm and the layer thickness *d*_1_ = 100 nm and *d*_2_ = 80 nm as illustrated in Figure 2b. Figure 2c,d shows the top and cross-sectional SEM images of the ZnO thin film on the porous silicon substrate sample (ZS1). We can see that the ZnO thin film was closely connected with the PS substrate and no clearance can be found in the interface. This may be due to the partial filling of the ZnO thin film in the pores. The ZnO film obtained after annealing at 700°C (corresponding to the sample ZS1-A) reveals the formation of labyrinth patterns, and the composite is composed of numerous spherical ZnO nanocrystals emerging in a network of pores as Figure 2e,f shows. The labyrinth patterns may be caused by the ZnO film, deposited on the PS substrate acting as a transparent coating on top of the porous structure. The air present in the pores is sealed up, and during the heating process of the substrate at 700°C, it starts to escape resulting in film stress and the formation of the crests, therefore the labyrinth patterns [20].

To optically characterize the composite, the luminescent properties of ZnO/PS structures were studied before and after annealing. Generally, all the characterized ZnO thin films exhibit two bands, one centered at 380 nm and the second one around 520 nm. The spectral position of the peak at 380 nm (3.27 eV) is attributed to the near-band edge excitonic recombinations in ZnO films [21], whereas the blue-green emission band peaking at 520 nm (2.38 eV) has been reported as the most common band for ZnO [22], typically attributed to the non-stoichometric composition of ZnO (defects mainly due to oxygen vacancies) [23].

**Figure 3:**
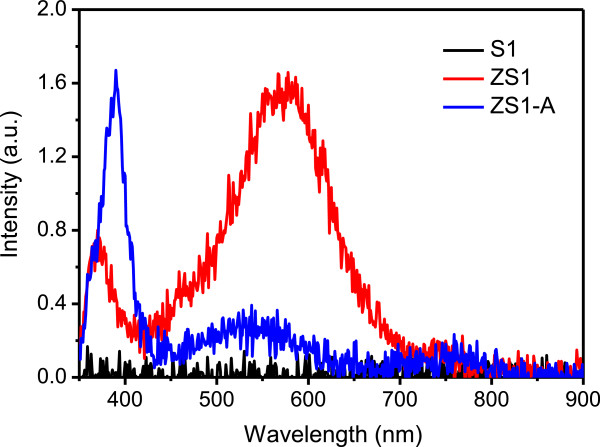
Photoluminescence spectra of porous silicon substrate (S1) and PS-ZnO composites before (ZS1) and after (ZS1-A) annealing at 700°C.

PL spectra of PS and ZnO/PS structures are shown in Figure 3. The PL spectrum of the sample ZS1 (corresponding to the ZnO/PS structure before annealing) shows emission in the UV region around 372 nm, characteristic of the near-band edge excitonic recombinations in the ZnO film [21]. The PL emission in the visible region could be attributed to the radiative recombination of the delocalized electron close to the conduction band with a deeply trapped hole in the zinc and oxygen vacancies (*V*_Zn−_, *V*_o+_) and oxygen centers (O_i_), respectively [21]. After annealing, the emission from the composite (ZS1-A) enhances in the UV region accompanied with a decrease in the visible range. The emission in the visible region is mainly due to deep-level defects (such as oxygen vacancies). The ratio of UV to visible emission has been considered as a key criterion to evaluate the crystalline quality. Consequently, a strong UV emission and weak green emission from ZnO could be attributed to the good crystalline quality of the ZnO film which is not the case before annealing. The deep-level emission is usually related to structural defects and impurities; however, the structural defects depend on lattice mismatch [24]. The PL emission band around 531 nm (2.3 eV) is associated with the radiative recombination of photogenerated holes with single ionized charge of specific defects such as oxygen vacancies or Zn interstitials [25-27].

**Figure 4:**
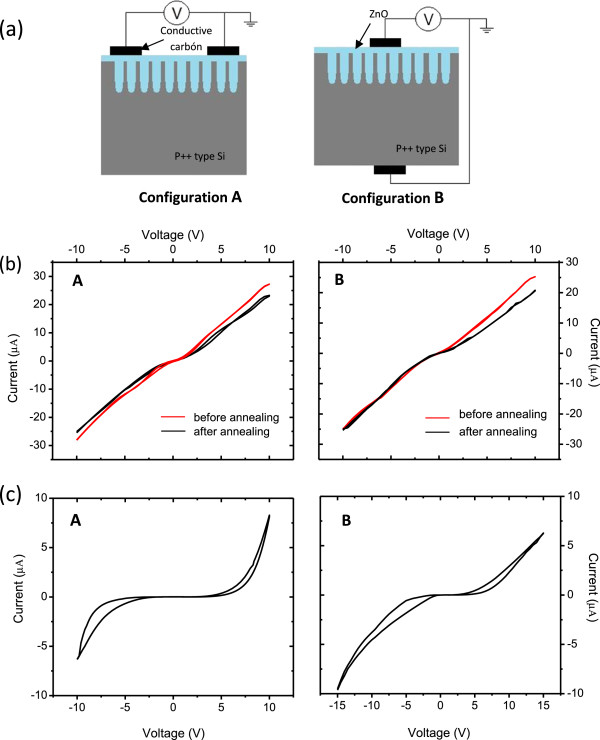
**Current-voltage ( *I *- *V *) characterization. (a)** Schematic of lateral (A) and transversal (B) measurements for the same sample. **(b)** ZnO over crystalline Si before and after annealing. **(c)** ZnO-mesoPS composite after annealing. Left- and right-hand side figures correspond to the configurations A (lateral) and B (transversal), respectively.

Figure 4a shows schematics of lateral (A) and transversal (B) configurations of the electrodes for current-voltage (*I*-*V*) characterization. Two types of configurations (lateral and transversal) for *I*-*V* characterization were analyzed in order to provide more information about the oxygen vacancies' diffusion paths. ZnO deposited on crystalline silicon and then annealed at 700°C was also characterized as a reference, before and after annealing (Figure 4b). Results illustrated in Figure 4b reveal a simple resistor-like behavior in both cases. Annealed ZnO-mesoPS composites were tested for memristive response for both configurations, and the current-voltage curves of our proposed device after annealing (Figure 4c) reveal the zero-crossing pinched hysteresis loop characteristic of memristive devices [2,28] in both cases. By analyzing the results in Figure 4c, we can clearly see a better curve symmetry for the lateral configuration (A), although some asymmetry is evident for both of them. Like a typical memristive device, the device state (*R*_off_ to *R*_on_) remains unaffected before a certain threshold voltage. In particular, for the case of lateral configuration, the memristive switching ratio from the high resistance state (HRS) to the low resistance state (LRS) at 7 V is 1.72 for the positive bias and 3.1 for the negative bias, which indicates a bipolar resistive switching.

In the literature, there are basically two possible mechanisms acting in the system for the transport of oxygen vacancies, which are responsible for the demonstration of memristive characteristics: (a) the filamentary conducting path [7-9] and (b) the interface-type conducting path [7]. The first one proposes that conductive and non-conductive zones in the oxide layers are created by the distribution of oxygen vacancies within the material due to its morphology and the applied bias voltage. The second one explains the resistive switching by the creation of conducting filaments made of oxygen vacancies across the dielectric material (ZnO) under an applied bias voltage. In the present study, the effect can be attributed to the fact that the use of porous silicon as a substrate increases the effective surface area (refer to Figure 2e; granular labyrinth patterns formed on the surface after annealing) and hence the oxygen vacancies in ZnO, which leads to the memristive behavior of the composite structure. Conductive channels (filamentary conducting paths) are formed within the ZnO layer and grain boundaries [7]. In both configurations, the presence of memristive behavior suggests that a suitable grain size can promote the diffusion of oxygen vacancies in any direction of the device.

## Conclusions

In this paper, the ZnO-mesoPS nanocomposite is demonstrated as a potential structure in the fabrication of memristive devices. Deposition of ZnO onto the mesoporous silicon substrate and post-annealing treatment resulted in the formation of regular labyrinth patterns with granular appearance. Mesoporous silicon as a substrate was found to promote the modification of ZnO grain size and consequently a significant enhancement of oxygen vacancies, which are responsible for resistive switching. Typical memristive behavior is demonstrated and analyzed. Future work is being carried out to study the tunability of the device as a function of substrate porosity/morphology.

## Competing interests

The authors declare that they have no competing interests.

## Authors' contributions

LM and OO carried out all the experimental work. VA and YK conceived the experiments. All the authors analyzed and discussed the results to structure and prepare the final version of the paper. All authors read and approved the final manuscript.

## Authors' information

LM and OO are PhD and M. Tech students, respectively, in a material science and technology program in a research institute (CIICAp-UAEM) in Cuernavaca. YK is a postdoctoral fellow in UNAM. VA is working as a professor-scientist in CIICAp-UAEM.
